# Neoplastic Cells are the Major Source of MT-MMPs in *IDH1*-Mutant Glioma, Thus Enhancing Tumor-Cell Intrinsic Brain Infiltration

**DOI:** 10.3390/cancers12092456

**Published:** 2020-08-29

**Authors:** Ina Thome, Raphael Lacle, Andreas Voß, Ginette Bortolussi, Georgios Pantazis, Ansgar Schmidt, Catharina Conrad, Ralf Jacob, Nina Timmesfeld, Jörg W. Bartsch, Axel Pagenstecher

**Affiliations:** 1 Departments of Neuropathology, Philipps University Marburg, 35043 Marburg, Germany; Ina.thome@staff.uni-marburg.de (I.T.); raphael.lacle@googlemail.com (R.L.); voss.andreas@klinikum-oldenburg.de (A.V.); bortolus@med.uni-marburg.de (G.B.); Georgios.Pantazis@ksgr.ch (G.P.); Catharina.Conrad@uth.tmc.edu (C.C.); 2 Departments of Pathology, Philipps University Marburg, 35043 Marburg, Germany; ansschmi@med.uni-marburg.de; 3 Departments of Clinical Cytobiology and Cytopathology, Philipps University Marburg, 35037 Marburg, Germany; jacob@staff.uni-marburg.de; 4 Department of Medical Informatics, Biometry and Epidemiology, Ruhr University Bochum, 44780 Bochum, Germany; Nina.Timmesfeld@rub.de; 5 Departments of Neurosurgery, Philipps University Marburg, 35043 Marburg, Germany; jbartsch@med.uni-marburg.de; 6 Centre for Mind, Brain, and Behaviour, 35032 Marburg, Germany

**Keywords:** matrix metalloproteases, glioma, isocitrate dehydrogenase, organotypic cell invasion assay, invasion

## Abstract

**Simple Summary:**

Most primary brain tumors infiltrate the surrounding brain even before the time of diagnosis and therefore cannot be removed completely. Matrix metalloproteases can degrade the extracellular proteins of the brain and thereby allow for the brain infiltration of glioma cells. Here, we demonstrate that tumor cells are the major source of several metalloproteases and as such responsible for the malignant behavior of gliomas. Our findings suggest that, controlling metalloproteases might be a promising therapeutic avenue in the treatment of glioma.

**Abstract:**

Tumor-cell infiltration is a major obstacle to successful therapy for brain tumors. Membrane-type matrix metalloproteinases (MT-MMPs), a metzincin subfamily of six proteases, are important mediators of infiltration. The cellular source of MT-MMPs and their role in glioma biology, however, remain controversial. Thus, we comprehensively analyzed the expression of MT-MMPs in primary brain tumors. All MT-MMPs were differentially expressed in primary brain tumors. In diffuse gliomas, MT-MMP1, -3, and -4 were predominantly expressed by IDH1^mutated^ tumor cells, while macrophages/microglia contributed significantly less to MT-MMP expression. For functional analyses, individual MT-MMPs were expressed in primary mouse p53^−/−^ astrocytes. Invasion and migration potential of MT-MMP-transduced astrocytes was determined via scratch, matrigel invasion, and novel organotypic porcine spinal slice migration (OPoSSM) and invasion assays. Overall, MT-MMP-transduced astrocytes showed enhanced migration compared to controls. MMP14 was the strongest mediator of migration in scratch assays. However, in the OPoSSM assays, the glycosylphosphatidylinositol (GPI)-anchored MT-MMPs MMP17 and MMP25, not MMP14, mediated the highest infiltration rates of astrocytes. Our data unequivocally demonstrate for the first time that glioma cells, not microglia, are the predominant producers of MT-MMPs in glioma and can act as potent mediators of tumor-cell infiltration into CNS tissue. These proteases are therefore promising targets for therapeutic interventions.

## 1. Introduction

Diffuse infiltration of the surrounding brain tissue is a hallmark of diffuse astrocytoma and glioblastoma, and regularly prevents the complete resection of these tumors [1]. Moreover, these tumors are relatively insensitive to radiation therapy and chemotherapy (for review, see Reference [2]). Despite the substantial efforts that have been undertaken to better understand glioma biology, these tumors still are generally fatal (for review, see Reference [3]). Since the migration of glioma cells is crucial to the malignant behavior of these tumors, the factors that confer this potential have been extensively studied [4]. Some of the protease members of the matrix metalloprotease (MMP) family have been shown to enhance glioma cell mobility [5]. About 25 years ago, there was much hope that MMP inhibition might be of therapeutic value in treating glioma patients. The broad-spectrum MMP inhibitors used at that time, however, showed severe side effects and had to be abandoned for glioma treatment (for review, see Reference [6]). Thus, detailed knowledge is required to understand the roles of individual MMPs in each tumor entity. Recently, the membrane-bound members of the MMP family, the MT-MMPs, have become a focus of tumor biology research. Four members of this family, MMP14, -15, -16, and -24, are membrane-bound by a transmembrane domain, while MMP17 and MMP25 are fixed to the membrane by a glycosylphosphatidylinositol (GPI) anchor [7]. MT-MMPs have pleiotropic effects that include the degradation of components of the extracellular matrix (ECM), the activation of soluble MMPs, and the shedding of ectodomains of various membrane-bound proteins (for review, see Reference [8]). The latter activity includes the (auto)degradation of themselves and other membrane-bound metalloproteases. All six members of the MT-MMP family are expressed in gliomas [9,10,11,12,13]. The effects of different MT-MMPs on the biological properties of gliomas, however, are not clear. MMP14 was the first MT-MMP discovered, and is the most extensively studied to date [14]. The complex of MMP14, TIMP2, MMP2 and a_v_ß_3_ integrin leads to localized MMP2 activation and increases glioma cell migration in vitro [15]. By cleaving tissue transglutaminase (tTG), MMP14 alters the migratory potential of U251 glioma cells, producing increased migration on collagen and decreased migration on fibronectin [16]. MMP14 enhances the proliferation and migration of human U251 glioma cells and elevates the expression of vascular endothelial growth factor (VEGF) in vitro [17]. In vivo, MMP14-transfected U251 cells formed larger and better vascularized tumors than control cells when subcutaneously transplanted into nude mice [17]. In a seminal study, Belien et al. demonstrated that MMP14 enables 3T3 fibroblasts to spread and migrate on inhibitory CNS membrane protein extracts, and to migrate in rat optic nerve explants [18]. In a later report, it was demonstrated that microglial MMP14 enables murine GL261 glioma cells to migrate in murine organotypic brain-slice cultures [19]. Much less is known about the role of the other MT-MMPs in glioma biology. The overlapping substrate panels of MT-MMPs, e.g., MMPs15, -16, and -24 are pro-MMP2 activators, suggest that these too might enhance the migration and infiltration of glioma cells; indeed, MMP15 and MMP16 have been demonstrated to enhance glioma cell migration in vitro. The GPI-anchored human MMP17 [20] is expressed in various glioma cell lines [12] and digests a number of ECM constituents as well as TNF [21]. Similarly to MMP17, the second GPI-anchored MT-MMP, MMP25 [22], digests a number of ECM components, including myelin basic protein and several chemokines, but not MMP2 (for review, see Reference [7]. MMP25-degraded vimentin has strong chemotactic and phagocytosis-enhancing effects on macrophages, indicating a role of MMP25 in the resolution of resorptive lesions (for review, see Reference [7]). Importantly, MMP25 did not enhance the migration of a carcinoma and a sarcoma cell line through type I collagen [23]. Significant levels of MMPs16, -17, and -24 are constitutively expressed in the normal CNS [7,20]. Furthermore, MMP17 is produced during embryonal and postnatal brain development [24,25]. MMP24 is located in differentiated neurons and regulates axonal growth and cadherin cleavage [26].

Given their substrate spectrum and their elevated expression in gliomas, all MT-MMPs may enhance the migration of glioma cells. In order to better understand the roles of the different MT-MMPs in glioma biology, we analyzed MT-MMP gene expression and their cellular sources in various primary brain tumors. We then investigated the effects of individual MT-MMPs on the migration of primary astrocytes in various substrates, including via a novel CNS infiltration assay termed organotypic porcine spinal slice migration (OPoSSM) assay, which is an in-vivo-like, laboratory-animal-saving test system that allows for the determination of white-matter infiltration by genetically altered cells. 

## 2. Results

### 2.1. MT-MMP Expression in Human Primary Brain Tumors

**Figure 1:**
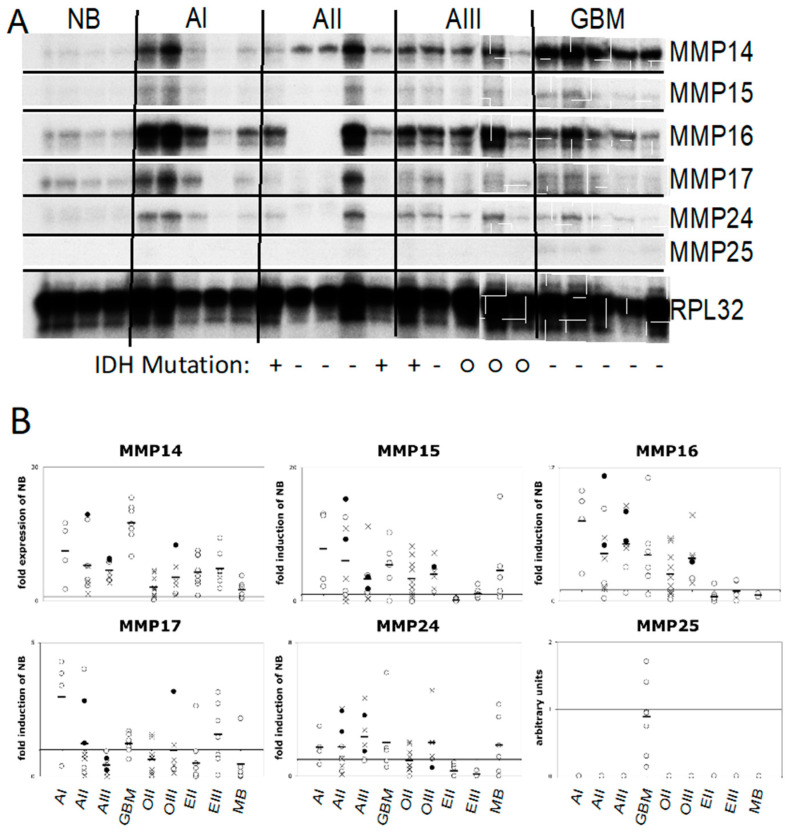
Gene expression of MT-MMP14, -15, -16, -17, -24 and -25 in 67 primary brain tumors. (**A**) A multiprobe RPA was established to determine the RNA amounts of the six hMT-MMP genes. *Ribosomal protein L32 (RPL32*) served as a loading control. Representative samples of normal brain (NB), piloctic astrocytoma (AI), diffuse astrocytoma (AII), anaplastic astrocytoma (AIII), and glioblastoma (GBM) are shown. The IDH mutation status of diffuse gliomas is indicated below the RPA image: + either IDH1 or IDH2 mutated; − IDH wild-type; ○ IDH status not known. (**B**) Levels of MT-MMP gene expression in different tumor types expressed relative to the level in normal brain tissue, diffuse oligodendroglioma (OII), anaplastic oligodendroglioma (OIII), ependymoma (EII), anaplastic ependymoma (EIII), and medulloblastoma (MB). (✕ diffuse gliomas or glioblastoma with a mutation in the IDH1 or IDH2 genes, ○ wild-type for either IDH, ● IDH status not determined).

We determined the expression of all six *MT-MMPs*, using RPA with probe sets developed for this study, in nine different brain tumor entities and grades (a total of 67 tumors; Figure 1A). All six *MT-MMPs* were expressed in primary brain tumors, and *MMP16* had the highest absolute RNA levels, followed by *MMP14*. Lower expression levels were observed for *MMP15*, -17, and -24, while *MMP25* was detected in glioblastoma only. All *MT-MMPs* were differentially regulated in the brain tumors tested (Figure 1B). The highly infiltrative astrocytomas, oligodendrogliomas, and glioblastomas showed strong upregulation of *MMP14*, *-15*, and *-16*, while there was only moderate upregulation of *MMP24* and no significant regulation of *MMP17*. In line with their more circumscribed growth, ependymomas showed increased expression of *MMP14* only, while the remaining five *MT-MMPs* were downregulated as compared to normal brain tissue. Interestingly, pilocytic astrocytoma was the tumor type with the strongest upregulation of *MMP15*, *-16*, and *-17*, making this tumor the maximum overall *MT-MMP* expresser. Glioblastoma was the only tumor type that revealed detectable expression of small amounts of *MMP25*. Medulloblastomas had the lowest expression of *MT-MMPs* (Figure 1B). 

### 2.2. Cellular Source and Distribution of MT-MMP-Expressing Cells in Human IDH-Mutated Gliomas

**Figure 2:**
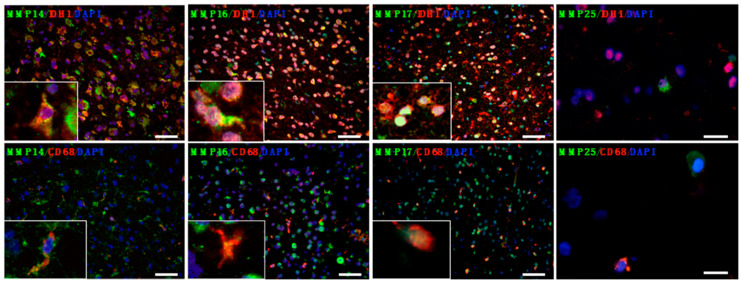
Characterization of MT-MMP-expressing cells in glioma tissue. Antibodies against mutated IDH1 identified tumor cells and anti-CD68 marked microglia/macrophages. Double staining with the respective MMP antibodies allowed for the identification of MMP-producing glioma cells and microglia/macrophages, respectively. DAPI was used to mark all nuclei and was used to calculate the total cell numbers in the slides. Each figure depicts overlays of three individual photographs taken of the stains using the respective filter cubes. (Scale bars for MMP14, -16, -17: 50 µm; scale bars for MMP25: 20 µm). Representative samples of diffuse or anaplastic astrocytoma are shown. Insets in the lower left show the triple labeling at higher magnification.

To differentiate MMP-expressing tumor cells from resident brain cells and activated microglia/macrophages, we assessed MT-MMP immunoreactivity in IDH1-mutated gliomas (four AII, five AIII, and four OII), because tumor cells bearing this mutation could be clearly identified using a specific IDH1 R132H antibody. In conjunction with the respective MT-MMP antibodies, it was possible to unequivocally identify MMP-expressing tumor cells (Figure 2). Possibly correlating with the very low RNA expression levels observed, MMP15 and MMP24 were not detectable by immunohistochemistry in glioma tissue. 

**Figure 3:**
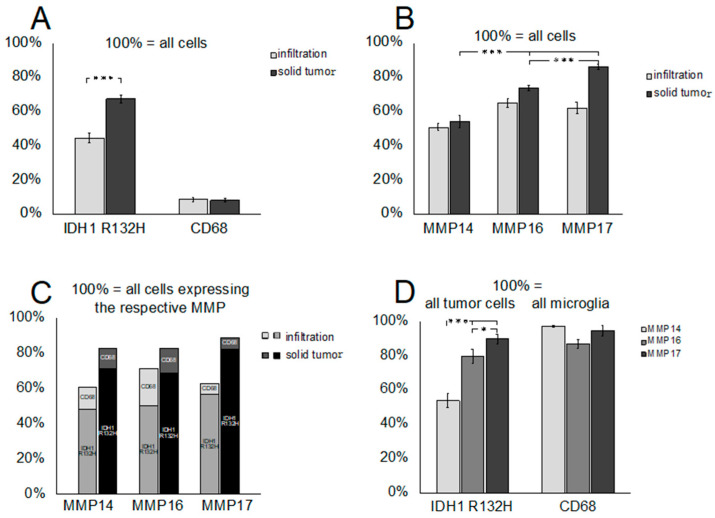
Cellular composition of IDH1-mutant gliomas (four AII, five AIII, and four OII). (**A**) IDH1-R132H-immunoreactive (IR) cells were more frequent in the solid portion of the tumor than in the infiltration zone, and presented a significantly higher percentage of cells than CD68-IR microglia/macrophages. (**B**) The individual MMPs were produced by different proportions of the cells, with MMP14 expressed by about 50% of cells, and MMP17 by 60% of cells in the infiltration zone and over 80% in the solid portion of the tumor. (**C**) Tumor cells constituted the largest fraction (50–80%) of MMP-expressing cells, while macrophages/microglia accounted for 5–20% of MMP-producers. (**D**) While nearly all CD68-IR microglia/macrophages produced all three MMPs, only 50%, 80%, and 90% of IDH-mutant tumor cells showed expression of MMP14, MMP16, and MMP17, respectively. (∗ *p* < 0.05; ∗∗∗ *p* < 0.001, *t*-test.).

Using DAPI stain as a marker for all nuclei and the antibody against mutated IDH1, we observed that tumor cells accounted for 60–75% of all cells in the solid portion of the tumor and significantly less (approximately 45%) in the infiltration zone, reflecting the higher percentage of constitutive CNS cells in the infiltration zone than in the solid portion of the tumor (Figure 3A). CD68-immunoreactive macrophages/microglia accounted for less than 10% of all cells in both the infiltration zone and the solid portion of the tumor (Figure 3A). The fraction of cells that produced MT-MMPs showed differences with regard to the particular MMPs and the location (infiltration zone vs. solid part of the tumor): 51% vs. 54% were positive for MMP14, 65% vs. 74% produced MMP16, and 62% vs. 86% expressed MMP17 (Figure 3B). About 50% of MMP-producing cells in the infiltration zone and 68–82% in the solid portion of the tumors were IDH1-mutated glioma cells. Microglia/macrophages amounted to 6–20% of MMP-expressing cells (Figure 3C). While the vast majority (86–97%) of microglia showed expression of individual MT-MMPs, we observed significant differences in the proportions of MMP expression in tumor cells: MMP14 was produced by 54%, MMP16 by 80%, and MMP17 by 90% of tumor cells (Figure 3D). This contrasted sharply to pilocytic astrocytomas, in which these MMPs were predominantly expressed in prominent vascular proliferations of these tumors (Figure S6).

Taken together, our findings demonstrated that in diffuse IDH-mutated gliomas, tumor cells represent the major sources of MMP14, -16, and -17, providing a rationale for functional analyses of MT-MMPs in glioma cells.

### 2.3. Characterisation of Virally Transduced Astrocytes 

Primary astrocytes showed constitutive expression of *mMMP14* and *mMMP16* (Figure S1A, Lanes 1 and 2, arrowheads). Transduced astrocytes showed strong expression of the respective *hMT-MMPs* (Figure S1B). The expression of *hMT-MMP* did not alter *TIMP* expression in the transduced astrocytes (Figure S1C). In contrast to normal brain tissue, which showed no constitutive expression of *TIMP1* and strong expression of *TIMP4*, we observed a strong band corresponding to *TIMP1* in all astrocyte lines, indicating an activated state of the astrocytes in culture, and no expression of *TIMP4*. All transduced astrocytes revealed strong EGFP fluorescence. EGFP was expressed under control of an IRES in the MIGR1 Vector (Figure S2A). Densitometry demonstrated comparable fluorescence levels of the various transduced astrocytes indicating comparable expression of the respective MT-MMPs (Figure S2B,C). Western blotting revealed strong bands for MMP14, MMP16, MMP17, and MMP25 in protein extracts from astrocytes transduced with the respective vectors (Figure S3, complete blots Figure S7). None of the commercially available antibodies tested produced signals for MMP15 and MMP24 on immunoblots. Control cells contained minor amounts of MMP14 and MMP17, indicating cross-reactivity of the antibodies with the respective murine MMPs, while MMP16 and -24 were not detectable (Figure S3). Immunocytochemistry revealed no or only faint staining in astrocytes transduced with the control vector (Figure S4A,C,E,G,I,K); strong cytoplasmic and surface staining was obtained for MMP14–17 and -24, while MMP25 revealed only weak signals (Figure S4B,D,F,H,J,L). Taken together, these data demonstrated that the transduced astrocytes expressed the respective proteases. 

### 2.4. Gelatin Zymography

Since at least four MT-MMPs can activate pro-MMP2, we analyzed the amount and activity of MMP2 in the supernatants of transduced astrocytes (Figure S5). In supernatants of control astrocytes as well as MMP-transduced astrocytes, the majority of gelatinolytic activity migrated at the lower molecular weight, representing activated MMP2, while only low levels of pro-MMP2 were detected (Figure S5, upper band). Transduction with *MT-MMP* genes did not induce further activation of pro-MMP2. This finding sharply contrasted with the result for supernatants of the human glioma cell line U87, which showed a strong band of pro-MMP2 and much less active MMP2. Incubation of U87 supernatants with transduced astrocytes resulted in an increase of the MMP2 activation, indicating that the murine astrocytes expressing human MT-MMPs were able to digest human pro-MMP2). Together, p53^−/−^ astrocytes had a very strong constitutive MMP2 activation that was not further increased by transduction with any of the *hMT-MMPs*. Taken together, these results indicate that the increased migration of astrocytes, as demonstrated in the migration assays shown below, was not mediated by MMP2.

### 2.5. The Type of Migration Assay Fundamentally Affects the Extent of the MT-MMP-Induced Increase of Astrocyte Migration 

#### 2.5.1. Scratch Wound Assay

**Figure 4:**
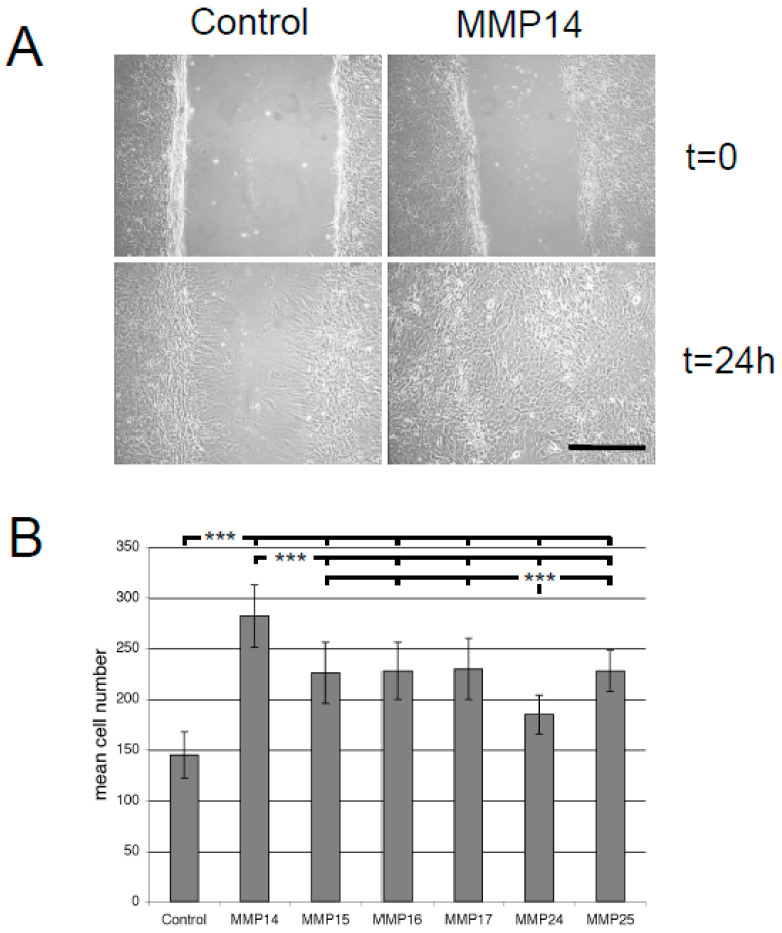
Cell migration determined by scratch assay. (**A**) Equal numbers of astrocytes were grown for two days in six-well plates. Twelve hours before scratching, cultures were washed with PBS and serum-free medium was applied. The scratch was applied using a pipette. The scratched area was photodocumented at five positions immediately after the scratch was applied and 24 h later. The number of astrocytes within the borders of the scratch was counted. (**B**) The graph shows the number of migrated astrocytes per image from two different experiments, each with three separately transduced astrocyte cultures (six biologically different replicates) for every MMP at *t* = 24 h. (∗∗∗ *p* ≤ 0.001, three-way ANOVA).

All genetic variants of astrocytes showed a strong capacity to migrate into a scratch applied to the culture (Figure 4A). We observed, however, strong differences between astrocytes transduced with the control vector and *MT-MMP*-transduced astrocytes. While 145 ± 23 (mean ± SD) control astrocytes migrated into the scratch, the number of migrating astrocytes for all *MT-MMP*-transduced astrocytes was significantly higher. MMP24 induced significantly fewer (185 ± 19) and MMP14 significantly more (281 ± 31) migrated cells than the other MMPs (Figure 4B) (*p* ≦ 0.001 three-way ANOVA).

#### 2.5.2. Matrigel Invasion Chamber

In repeated experiments, we consistently observed only very small numbers of transduced astrocytes or none at all that migrated through the matrigel invasion chamber to the other side of the membrane, while control experiments with human U87 glioma cells revealed large numbers of transmigrating cells. In order to analyze this difference, we plated transduced astrocytes and U87 glioma cells on matrigel-coated and matrigel-free membranes. While the astrocytes spread out on the matrigel-coated membranes, we observed death of all astrocytes plated on the matrigel-free membranes. In contrast, U87 cells survived on matrigel-free as well as on matrigel-coated membranes. This finding demonstrated that the transduced primary astrocytes were able to survive on matrigel but not on the membranes. Therefore, the matrigel invasion chamber was unsuitable to assess the migration potential of transduced astrocytes.

#### 2.5.3. Organotypic Porcine Spinal Slice Migration (OPoSSM) Assay

**Figure 5:**
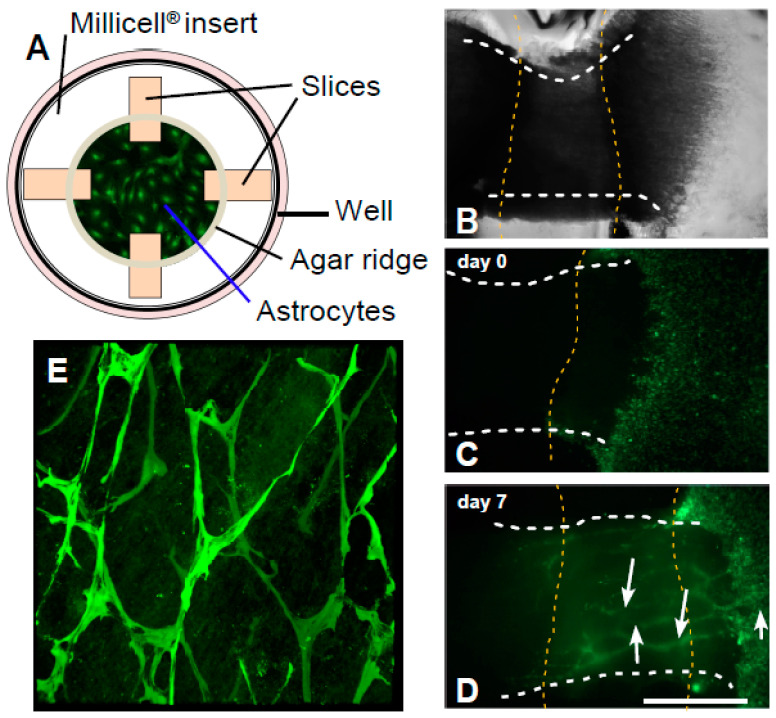
(**A**) Schematic depiction of the OPoSSM assay. Porcine spinal slices were positioned on a Milliwell membrane and a circular agarose wall was placed over the slices, thereby forming an inner and an outer chamber on the membrane. Astrocytes were placed into the inner chamber. (**B**) Agar (borders indicated by the yellow hatched lines) was placed over the spinal slices (longitudinal borders indicated by white hatched lines). Astrocytes were placed in front of the cut sections of spinal slices and allowed to migrate for 1 week. (**C**) Fluorescence of EGFP-expressing astrocytes right after application of the cells onto the membrane. (**D**) After one week of incubation, migrating astrocytes formed interlacing chains (white and yellow hatched lines: see legend to **B**). (**E**) Slices were fixed and immunohistochemical staining for EGFP was performed in order to enhance astrocytic EGFP fluorescence within the slices. Confocal microscopy was performed to allow the visualization of astrocytic chains at different depths of the slices. Original magnification 200 ×.

In order to assess the migration of transduced astrocytes in an authentic in-vivo-like system, we established the OPoSSM assay (Figure 5). Transduced control astrocytes and MT-MMP-expressing astrocytes were placed on Milliwell membranes in direct contact with the cut surfaces of spinal cord slices prepared from white matter and incubated for 1 week (Figure 5B–D). All genotypes of astrocytes showed a strong propensity to invade the spinal slices. The migrating astrocytes aggregated to interlacing chains within the slices (Figure 5D). Confocal imaging revealed that the chains assembled three-dimensional polygonal cage-like structures (Figure 5E, Video S1). A virtually identical migration pattern was observed for U87 human glioblastoma cells.

**Figure 6:**
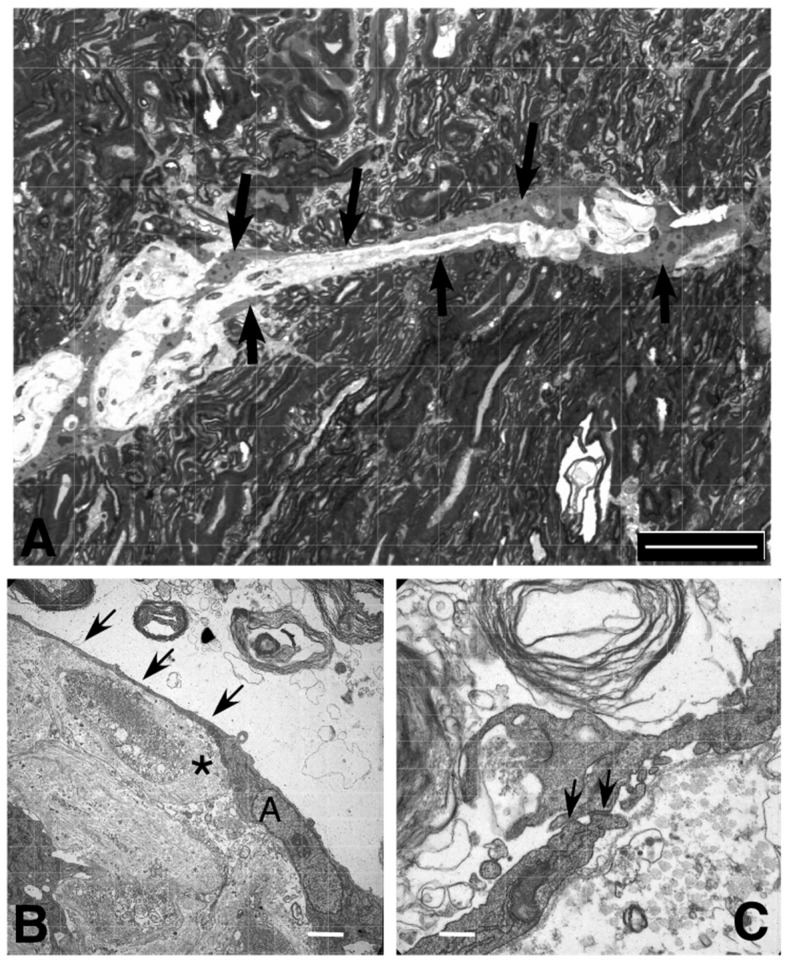
Light microscopy and ultrastructural analysis of resin embedded material. Slices were fixed in glutaraldehyde and embedded in plastic resin. (**A**) Semithin sections revealed that control and MMP-transduced astrocytes migrated predominantly along the capillaries (arrows; toluidin blue; scale bar: 50 µm). (**B**) Electron microscopy revealed astrocytes predominantly between the basal lamina of capillaries and the myelin of the white matter. Astrocytes produced very long, thin processes (arrows; asterisk: pericyte; A: nucleus of the astrocyte; scale bar: 2.5 µm) and (**C**) formed tight junctions with the processes of other astrocytes (arrows; scale bar: 400 nm).

Light-microscopic examination of resin-embedded material revealed that most invading astrocytes migrated along capillary basal laminae (Figure 6A). Ultrastructurally, there was only one spindle-shaped astrocyte that protruded two delicate long processes in both directions of the space between the vessel and the white matter (Figure 6B). The migrating astrocytes formed distinct contact areas with each other that presented as tight junctions (Figure 6C, arrows). 

**Figure 7:**
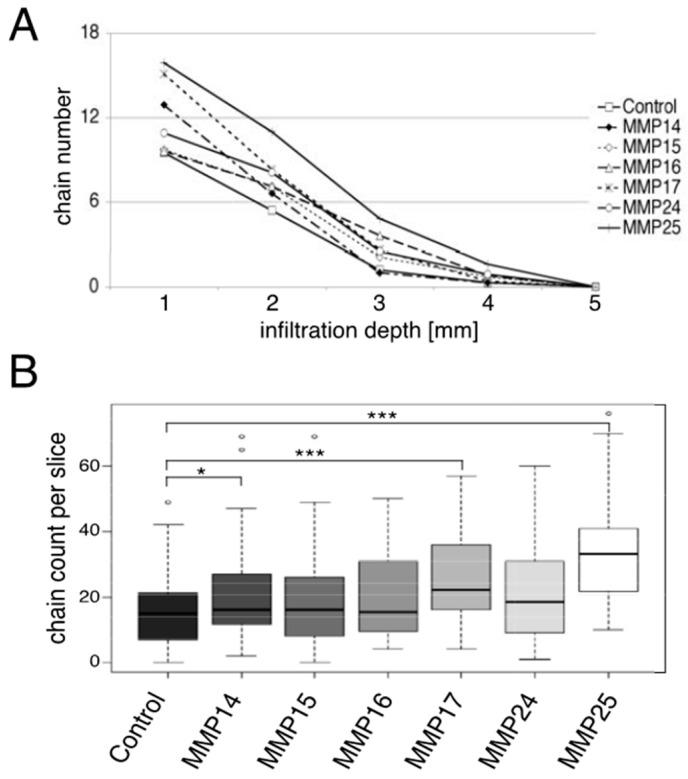
Migration of astrocytes as determined by the OPoSSM assay. Numbers of astrocyte chains in the spinal slices were counted every millimeter from the cut surface of the slices. The figure represents the results of two independent experiments with two different sets of transductions that were counted by four independent observers blinded to the genotype of astrocytes. (**A**) Number of astrocyte chains for every millimeter of infiltration depth. For better legibility, the standard error bars have been omitted. All MMP-transduced astrocytes showed increased infiltration into the spinal cord slices. (**B**) Total number of astrocyte chains per slice. ANOVA test showed significant differences between control astrocytes and astrocytes expressing MMP14, -17, and -25 (∗ *p* < 0.017, ∗∗∗ *p* ≤ 0.001).

The infiltration capacity of transduced astrocytes was assessed as the number of chains counted every millimeter from the edge of the slice along the whole cross-section. All *MMP-*transduced astrocytes showed a higher migration potential than control astrocytes. The analysis of chain numbers and depth of penetration, however, revealed a complex MMP-dependent pattern (Figure 7). The MMP14-expressing astrocytes showed increased chain numbers in the first two millimeters of the slice and comparable values to control astrocytes at greater depths. At three millimeters, there were significant chain numbers observed for astrocytes expressing MMP16, -17, -24, and -25 (Figure 7A). Testing for the parameters infiltration depth and chain number by ANOVA demonstrated statistical significance for enhanced migration by MMP14, MMP17, and MMP25 as compared to control astrocytes (Figure 7B). Interestingly, the two GPI-anchored MMPs, MMP17 and MMP25, showed the strongest infiltration-enhancing effect in the OPoSSM assay.

## 3. Discussion

The most frequent primary brain tumor types show diffuse invasion of the surrounding brain tissue, which frequently precludes complete resection [1]. Here, we investigated the expression levels and cellular sources of the six human MT-MMPs in various primary brain tumors, and analyzed their roles in the migration of primary astrocytes in different model systems. We observed differential expression patterns of all six *MT-MMPs* in the various tumor types investigated. Published data on the expression of *MT-MMPs* in glioblastoma, astrocytoma, and oligodendroglioma show considerable differences [9,10,12,27,28]. The TCGA platform, for example, shows upregulation of *MMP16* in the proneural type of glioblastoma only, the REMBRANDT database reports moderate upregulation in low-grade gliomas and glioblastoma (both retrieved from betastasis.com), while a recent report using a French dataset calculated an approximately 2-fold upregulation [28]. Concurrently, *MMP16* had the highest expression levels in NB and gliomas [28], a finding that we confirmed in the present study. In line with this, a recent paper used the TCGA data and found that expression levels of *MMP16* and *MMP17* in gliomas were in the top two of all cancers [13]. These apparently contradictory observations are due to the strong constitutive CNS expression of several *MT-MMPs*, particularly *MMP16*, *-17*, and *-24*. Since most reports present *MMP* expression as a multiple of the expression level in normal brain tissue, high constitutive expression causes low numbers of fold induction or even downregulation in glioma, notwithstanding that glioma cells produce significant amounts of the proteases. This explains the fact that these MMPs have not received the same consideration as MMP14 in glioma research. 

Here, we extended the analysis of *MT-MMP* expression to pilocytic astrocytomas, ependymomas, and medulloblastomas. While the low *MT-MMP* expression levels in ependymomas and medulloblastomas correlated well with their low infiltration tendency, we observed the overall maximum expression of *MMP14*, *-15*, *-16*, and *-17* in pilocytic astrocytoma. This apparent contradiction was explained by the strong expression of at least three of these MMPs in the prototypic vascular proliferations, while tumor cells showed no or only nuclear expression.

In diffuse gliomas, the majority of MT-MMP-producing cells were IDH-mutated tumor cells, while CD68-immunoreactive microglia/macrophages constituted a much smaller proportion. Glioma cells and macrophages accounted for 80–90% of MT-MMP-positive cells in the solid portion of the gliomas, and thus there was only a small number of uncharacterized MT-MMP producers. Our findings are in line with several reports of MMP14 expression in glioma cells and most glioma cell lines [29,30]. They are in contrast, however, to the results of a study in which 28 to 91% of MMP14-expressing cells in diffuse gliomas and glioblastoma were identified as Iba1-immunoreactive microglia [19]. A possible explanation for this discrepancy might be that these observations were based on limited material of a tissue array and that the numbers of MMP14-expressing tumor cells were not determined separately [19]. Our approach unequivocally identified for the first time MT-MMP-producing microglia/macrophages and tumor cells and highlighted the preeminence of tumoral microglial MMP-overexpressers in diffuse glioma. 

We then determined the functional roles of glioma-derived MT-MMPs in murine primary p53^−/−^ astrocytes, because genetic alteration of *Tp53* is a common finding in low-grade astrocytoma and secondary glioblastoma [31]. All assays were performed with early passages of transduced p53^−/−^ astrocytes, because malignant transformation of these cells has been demonstrated after prolonged passaging in cell culture [32]. 

Most MT-MMPs can activate pro-MMP2 (for review, see Reference [7]). Therefore, we used gelatin zymography to investigate the pro-MMP2 activation in supernatants from transduced astrocytes. In contrast to many published studies that examined glioma cell lines [33], we observed that the largest portion of MMP2 in supernatants from primary astrocytes was activated. Expression of hMT-MMPs did not further increase the MMP/pro-MMP ratio. Supernatants from U87 glioma cells, in contrast, contained more pro-MMP2 than active MMP2. This unexpected finding was explained by the constitutive expression of mMMP14, mMMP16, and low levels of mMMP25 in primary astrocytes. Importantly, this finding demonstrated that the enhanced migration of transduced astrocytes was not caused by activation of MMP2.

A plethora of factors enhance the potential of astrocytoma cells to diffusely infiltrate the surrounding brain tissue [4,34]. An early report demonstrated that MMP14 enables 3T3 fibroblasts to migrate along the white matter, while control fibroblasts do not spread along myelin [18]. In glioma cells, MMP14 activity in the lamellipodia mediates glioma migration [29]. A competing idea states that microglial MMP14 enables glioma cell infiltration. This work, however, was performed with MMP14-negative glioma cell lines, which showed cell death upon forced expression of MMP14 and therefore may apply to this rather rare situation only [19]. Here, we investigated the potential of MT-MMPs to enhance the migration of astrocytes in different in vitro assays. In scratch assays, all MT-MMPs enhanced astrocyte migration compared to control astrocytes and MMP14 was the most potential mediator of migration, showing nearly a two-fold increase of migrated astrocytes when compared to control astrocytes. In contrast to U87 glioma cells, we did not observe migration of transduced primary astrocytes in matrigel transwell chambers. A literature search revealed that the matrigel membrane is toxic to primary astrocytes [35]. Therefore, this assay was not suitable for determining the migratory potential of primary astrocytes. The factor(s) that contribute to this difference between primary astrocytes and glioma cell lines are not clear. Nevertheless, U87 cells and other established cell lines that show transmigration in matrigel assays migrate poorly in CNS tissue, which is a major limitation of the matrigel model. We therefore developed the OPoSSM assay, an in vitro model that more closely emulates the in vivo situation. All genetic variants of astrocytes readily entered the slices and migrated predominantly along the capillaries between the vascular basal lamina and the myelin of the white matter. This localization resembles that seen in fetal astrocytes implanted into the rat brain as well as that of glioma cells, which has been attributed to a particular permissiveness for migration of this compartment [36,37]. Moreover, contact with the vascular basal lamina is a physiological localization for astrocytes [38], which also explained our observation that the astrocytes regularly merely formed single layers around the basal lamina of the capillaries. In the present study, the migrating astrocytes had a bipolar (piloid) shape that resembled radial glia. This feature might reflect the potential of mature astrocytes to transform into transitional radial glia in the lesioned cerebral cortex [39]. Thus, the OPoSSM assay recapitulated important infiltration characteristics of primary brain tumors. 

All MT-MMPs enhanced the migration of astrocytes in the OPoSSM assay. The most potent mediators of astrocyte migration were MMP14, -17, and -25. Interestingly, MMP14 showed higher numbers of migrating astrocytes than the control in only the first two millimeters from the edge of the slice, while the GPI-anchored MMP17 and MMP25 had the maximum migration-enhancing effect on astrocytes with regard to both chain number and penetration depth. Since MT-MMPs have different substrate specificities and activities, these different effects on migration might be due to a direct effect of a particular MT-MMP on the migration of astrocytes, or an indirect effect that is mediated by downstream factors that are activated by this particular MT-MMP [40]. A likely candidate would be MMP2, because this gelatinase is activated by most of the MT-MMPs and can enhance the migration of different cell types, such as 3T3 fibroblasts and glioma cells [18,29,41]. The astrocytes used in our model showed mostly activated MMP2 in culture supernatants, and we did not observe further activation by any of the MT-MMPs. Therefore, it is unlikely that active MMP2 mediated the MT-MMP-induced enhancement of astrocyte migration in our migration and invasion models. One could rather argue that the high amount of active MMP2 secreted by the astrocytes used here led to the constitutively high migration potential of these cells, thereby dwarfing the effect of the transduced MT-MMPs. It is not clear why the GPI-anchored MMP17 and MMP25 had the strongest effect on astrocyte migration. MMP17 digests ECM substrates such as gelatin and is, among others, an activator of proTNF and of ADAMTS4 [21]. Active ADAMTS4 digests several ECM constituents including the brain-specific brevican, and enables brain invasion of 9L rat gliosarcoma cells [42]. This mechanism could be in action in our model and might be relevant for human disorders, since ADAMTS4 is expressed in human glioblastoma [43]. 

## 4. Materials and Methods

### 4.1. Tissue Samples

Brain tumor tissue samples were obtained from patients who underwent neurosurgery for tumor resection. Normal neocortical brain tissue samples were removed in the course of surgical hippocampectomy for refractory epilepsy. Tissues were dissected, one portion was immediately snap-frozen in liquid nitrogen and stored at −80 °C, and the other portion was fixed in 4% formaldehyde and embedded in paraffin (FFPE). Histological diagnosis was made according to the current WHO criteria [1]. Four specimens of pilocytic astrocytoma (WHOgrade I; AI), 9 fibrillary astrocytomas (WHO grade II; AII; four IDH-mutated (mut), three wild-type (wt), two not determined (n.d.)), 7 anaplastic astrocytomas (WHO grade III; AIII; three mut, two wt, two n.d.), 9 glioblastomas (WHO grade IV; GBM; all wt), 10 oligodendrogliomas (WHO grade II; OII; nine mut, one wt), 6 anaplastic oligodendrogliomas (WHO grade III; OIII; five mut, one n.d.), 9 ependymomas (WHO grade II; EII), 7 anaplastic ependymomas (WHO grade III; EIII), 6 medulloblastomas (MB; WHO grade IV), and 5 normal brain samples (NB) were included in this study. Immunohistochemical analysis was performed on FFPE material from 13 IDH1-mutated gliomas (4 AII, 5 AIII, and 4 OII). IDH mutation status of diffuse gliomas (astrocytomas and oligodendrogliomas) as well as of GBM was determined via an antibody against IDH1 R132H. Tumors that did not stain with this antibody were analyzed by pyrosequencing for mutations in the IDH1 Codon 132 and IDH2 Codon 172, as described [44]. The diagnosis of oligodendroglioma was based on histology only, because archival material was analyzed (oligodendroglioma NOS according to the current WHO classification). Informed consent for the use of the patient material was given by the patients and the study was approved by local ethics committees (University of Freiburg (320/01) and University of Marburg (185/11)).

### 4.2. RNase Protection Assay (RPA)

Total RNA was extracted with Trizol^®^ reagent (Invitrogen GmbH, Darmstadt, Germany). The expressions of the six human *MT-MMPs* were determined with a novel multiprobe RPA containing probes for the six human *MT-MMP* genes and a probe for the internal loading control, ribosomal gene RPL-32. The primers used for the generation of the MT-MMP RPA probes are specified in Table S1. The development of this probe set was performed as described previously [45]. In brief, the sequences of the six human (h) MT-MMPs were aligned (MegAlign, DNAStar, Madison, WI, USA) and primers were designed in order to amplify the unique sequence fragments specific to individual hMT-MMPs to be used as probes. Each probe was initially tested separately in order to verify the protected RNA. Subsequently, the multiprobe set was established. The RPA probe set for the detection of murine *MT-MMPs* was described previously [24]. The RPAs were performed as described previously [45]. Quantification was performed based on scanned autoradiographs, using ImageJ software as described previously [46]. Band density of the individual mRNAs was normalized to that of L32. The resulting *MMP* expression in individual samples was normalized to the mean value of the respective normal brain (NB) expression and plotted as fold induction of NB. 

### 4.3. Immunohistochemistry and Immunocytochemistry

IDH-mutant brain tumors were sectioned at 3 µm and autofluorescence was blocked with the MaxBlockTM Autofluorescence Reducing Reagent Kit (MaxVision Biosciencess Inc., Washington, WS, USA). Tumor cells were detected with an antibody against IDH1 R132H (H09, 1:50, DIANOVA, Hamburg, Germany) and macrophages/microglia were stained with an antibody against CD68 (PG-M1, 1:100, Agilent, Santa Clara, CA, USA). Total cell numbers were calculated using the number of 4′,6-diamidin-2-phenylindol (DAPI)-stained nuclei. Antibodies for the detection of MMPs were: MMP14: ab51074, 1:100, Abcam, Cambridge, UK; MMP16: C0268, 1:50, Assay bioTech, Fremont, CA, USA; MMP17: GTX29125, 1:100, Genetex, Irvine, CA, USA; MMP25: ab39031, 1:100, Abcam. For each tumor, five areas (0.12 mm^2^) of both the infiltration zone and the solid tumor were photographed thrice using three different filter cubes that allowed for the detection of the fluorescence of the secondary antibodies and DAPI. The number of stained cells was counted with the Cell Counter Plugin provided by the Fiji Software [47]. Astrocytes were grown on coverslips, washed in PBS, and fixed for 45 s in a 1:1 mixture of methanol and acetone at −20 °C. Subsequently, the cells were incubated O/N at 4 °C with the respective primary antibodies (MMP14: clone EP1264Y, dilution 1:20, Epitomics, Burlingame; CA, USA, MMP15: clone 130522, dilution 1:20; ^MM^P24: rabbit polyclonal, AB924, dilution 1:10 MMP25: clone 141825, dilution 1:20 all from R&D Systems, Wiesbaden, Germany; MMP16: rabbit polyclonal, ab 11622, dilution 1:40, Abcam; MMP17: rabbit polyclonal, 475934, dilution 1:40, Calbiochem, La Jolla, CA, USA). Matching Alexa-Fluor-labeled secondary antibodies (Invitrogen) were used to visualize bound primary antibodies.

### 4.4. Cells and Constructs

Primary astrocytes were prepared from newborn p53^−/−^ mice as described previously [48]. Briefly, newborn mice on a TP53^−/−^ background were sacrificed by decapitation, brains were removed aseptically, and the meninges were removed. Subsequently, the cortices were minced and the cells were triturated by passaging through a glass pipette. The cells were seeded on 10 cm plates in DMEM containing 10% FBS and 1% penicillin/streptomycin. The cDNAs for human *MMP14*, *-15*, *-16*, *-17*, *-24*, and *-25* (accession numbers: NM_004995, NM_002428, NM_005941, NM_016155, NM_006690, NM_022468, all a generous gift from the M. Seiki lab (Tokyo, Japan) were cloned into the MIGR1 vector and transfected into Phoenix-Eco cells [49]. Supernatants of these were used to transduce the primary astrocytes as described previously [48]. The successful transduction was detected via EGFP fluorescence of transduced cells.

### 4.5. Western Blotting

Astrocytes were grown to confluency, washed with PBS, and harvested with a cell scraper in RIPA buffer (pH 8.0) containing Complete^®^ protease inhibitor cocktail (Roche, Mannheim, Germany). Protein samples (50 µg) were dissolved in Laemmli buffer, separated by SDS-PAGE, and blotted onto a PVDF membrane (Immobilon, Millipore, Burlington, MA, USA). The following primary antibodies were used: MMP14, dilution 1:2000; MMP15, dilution 1:2000; MMP24, dilution 1:2000; and MMP25, dilution 1:2000, from the same suppliers as for immunocytochemistry; MMP16 (#109378S Genetex), dilution 1:1000; MMP17 (clone EP1270Y, Epitomics), dilution 1:1000. Bound primary antibodies were detected with secondary antibodies (goat anti-mouse or anti-rabbit POX, dilution 1:5000 (Jackson ImmunoResearch, Ely, UK) using a CCD camera (INTAS, Göttingen, Germany)**.** To assess equal loading in different lanes, the membranes were incubated with an antibody against GAPDH (MAB 374, dilution 1:4000, Chemicon/ Merck Millipore, Burlington, MA, USA) and processed as described above.

### 4.6. MMP Activity Assays

Gelatin zymography was performed as previously described with modifications [46]. Here, 20 µL supernatant samples from transduced murine astrocytes or from human U87 glioma cells incubated in FBS-free DMEM for 48 h were analyzed.

Scratch wound assays were performed in six-well plates in FBS-free medium. A scratch was applied using a pipette tip. The width of the scratch was documented for each experiment via five microphotographs at 10× magnification taken directly after applying the scratch and 24 h later. The number of astrocytes that migrated into the scratch was counted in all photomicrographs. For each MT-MMP or the control vector, six independent experiments were performed using three sets of independently transduced astrocyte cultures. 

Matrigel invasion assays were performed with a commercial kit (BD Biosciences, San Jose, CA, USA) as detailed by the manufacturer. Control and genetically altered primary astrocytes, or U87 human astrocytoma cells were incubated for 24 h with FBS as the chemoattractant in the lower chamber. Transmigrating cells were counted after staining with toluidine blue/PBS. 

### 4.7. Organotypic Porcine Spinal Slice Migration (OPoSSM) Assay

To determine the effect of the various MT-MMPs on the three-dimensional migratory potential of astrocytes in white matter, we developed a novel migration assay. Porcine spinal cords were obtained immediately after slaughter of the animals (a generous gift from Toni’s Wurstladen, Bauerbach, Germany). The white matter was dissected and 2 cm longitudinal specimens prepared. These were shock-frozen and stored at −80 °C. Organotypic slices (400 µm) were prepared from defrosted slices as described previously for cerebellar tissue [50]. Four slices were placed onto the membrane of a Millicell cell-culture insert (Millipore) and a circular ridge of sterile 1.5% agar was applied onto the slices and the membrane, separating an inner and an outer chamber (Figure 5A). Next, 10^5^ transduced astrocytes were placed in front of the inner-chamber end of each slice. After 7 days of incubation, slices were fixed in 4% buffered formaldehyde and immunofluorescence against EGFP was performed to enhance the visibility of invaded astrocytes [50]. Slices were permeabilized and blocked by incubation in 0.3% Triton-X100, 1% BSA in PBS O/N at 4 °C, and then the primary antibody (rabbit anti-eGFP PAB14326, dilution 1:5000, Abnova, Taipei, Taiwan) was applied and bound antibody was detected with the secondary antibody (Alexa Fluor 488 labeled goat anti-rabbit, A11008, dilution 1:1000, Invitrogen). Since the astrocytes formed interconnected strings in the slices, the number of EGFP-fluorescent strings was counted at 1, 2, 3, 4, and 5 mm from the inner edge of each slice. Three independent transductions were performed for each MT-MMP and the control vector. At least 18 slices were counted for each condition by four experimenters blinded to the genotypes of the astrocytes. Confocal images of fixed cells were acquired on a Leica TCS SP2 microscope using a 20× fluotar lens (Leica Microsystems, Wetzlar, Germany). For 3D reconstruction, fluorescent images of up to 25 layers were recorded over a range of 100 µm. The images were processed via 3D rendering using by the velocity software package (Improvision, Coventry, England).

For electron microscopy, the slices were fixed in 2% glutaraldehyde buffered in Sörensenbuffer, postfixed in osmium tetroxide, and embedded in plastic resin. Semithin sections (1 µm) were stained with toluidine blue; ultrathin sections were contrasted with lead citrate and uranyl acetate as described previously [51]. 

### 4.8. Statistics

Immunohistochemical findings for human gliomas were analyzed by a two-sided *t*-test. For analysis of the scratch assays, three-way ANOVA with the factors of individual MT-MMP, cell culture, and experiment were used to test for differences between MT-MMP groups. Pairwise comparisons between all MT-MMP groups and control were performed through Tukey-type linear contrast tests. Since infiltrating chains in the OPoSSM assay were counted repeatedly by different observers, a two-way ANOVA for repeated measurements with the factors of individual MT-MMP and infiltration depth was used to test for statistical significance between MT-MMP groups. For pairwise comparisons between the MT-MMPs and control, Tukey–type linear contrast tests were used. All analyses were performed with the statistical software R (www.r-project.org), version 2.15.0 and the package multcomp. *p*-Values less than 0.05 were considered statistically significant.

## 5. Conclusions

MT-MMPs are pleiotropic factors with a multitude of biological actions ranging from the activation of other MMPs and the shedding of membrane-bound proteins to cell migration. The roles and cellular sources of MT-MMPs in glioma have been a source of controversy. Using mutated IDH1 as a distinct identifier of glioma cells, we here showed for the first time that glioma cells and not microglia are the definitely predominant producers of MT-MMPs in diffuse glioma. Moreover, we demonstrated that astroglia-intrinsic MT-MMP production significantly contributes to the infiltrative and migratory capability of these cells in vitro. Our observations clearly illustrated the fundamental impact that the selected migration medium exerts on the migratory behavior of glioma cells. The novel OPoSSM assay described here is an animal-saving in vitro model that enables the investigation of cell migration in authentic CNS tissue. Lastly, although barely upregulated in brain tumors as compared to normal brain tissue, MMP17 is (1) the MT-MMP expressed by most glioma cells and (2) a potent enhancer of astrocyte migration in CNS tissue. Thus, the MT-MMP family represents a worthwhile target for the treatment of glioma. 

## Supplementary Materials

The following are available online at https://www.mdpi.com/2072-6694/12/9/2456/s1, Figure S1: Detection of human and murine MT-MMP mRNAs by RPA, Figure S2: Expression of enhanced green fluorescent protein (EGFP) in transduced astrocytes, Figure S3: Expression of human MMP protein in transduced astrocytes, Figure S4: Expression of MT-MMP proteins in transduced astrocytes, Figure S5: Gelatin zymography. Equal numbers of astrocytes were cultured for two days in serum-free medium, Figure S6: Immunohistochemical staining of MT-MMPs in pilocytic astrocytoma, Figure S7: Detailed information about Western blot in Figure S3, Table S1: Primers and probe length for the human MT-MMP probe set, Video S1: Tilting of z-stacks reveals the three dimensional lattice formed by infiltrating astrocytes.
