# Supplementary material for: Neoplastic Cells are the Major Source of MT-MMPs in IDH1-Mutant Glioma, Thus Enhancing Tumor-Cell Intrinsic Brain Infiltration
Source: Cancers (Basel). 2020 Aug 29;12(9):2456. doi: 10.3390/cancers12092456 (PMC7565296; doi:10.3390/cancers12092456)
Supplement: Supplementary file 1 [file cancers-12-02456-s001.pdf]

Supplementary Materials

Neoplastic Cells are the Major Source of MT-MMPs in IDH1 Mutant Glioma, Thus Enhancing Tumour Cell Intrinsic Brain Infiltration

Ina Thome, Raphael Lacle, Andreas Voß, Ginette Bortolussi, Georgios Pantazis, Ansgar Schmidt, Catharina Conrad, Ralf Jacob, Nina Timmesfeld, Jörg W. Bartsch and Axel Pagenstecher

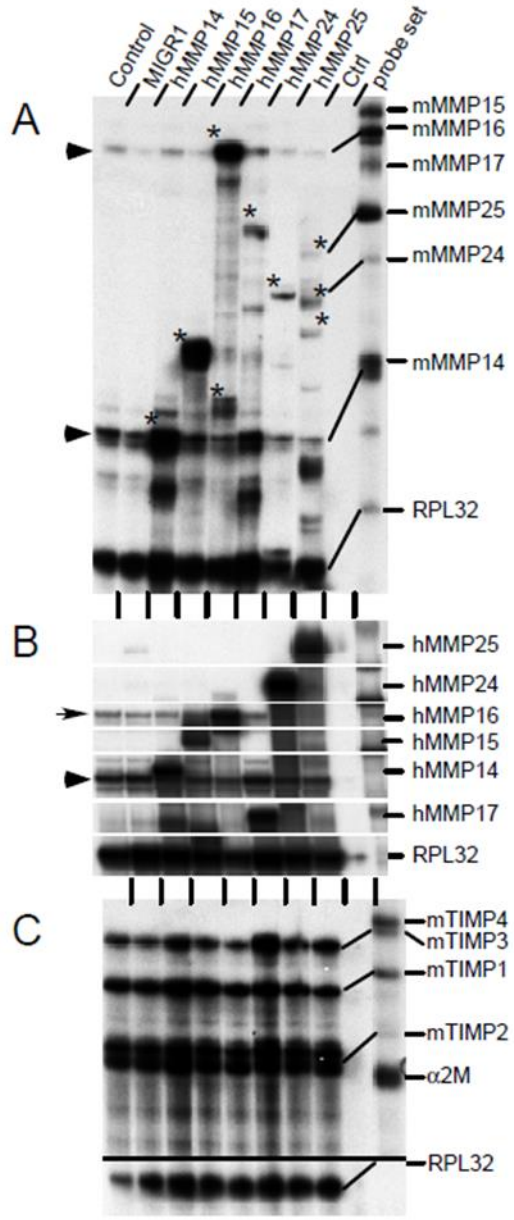

Figure S1. Detection of human and murine MT-MMP mRNAs by RPA. (**A**) Constitutive expression of murine MMP 14 and MMP 16 in primary astrocytes (arrowheads). The strong additional bands (asterisks) in several hMT-MMP transduced astrocytes are due to hybridization of the murine probe to the human RNA. For some MMPs such as MMP16 this band migrates at the proper molecular weight, in other cases such as MMP15 the protected portion of the hybridized murine probe is much shorter. (**B**) The transduced astrocytes expressed high levels of RNA of the respective human MMP

genes. The human probes for MMP 14 and MMP 16 show the constitutive expression of the murine homologues (arrow and arrowhead) 1 (**C**) Expression of murine TIMP genes was not altered by the expression of human MT-MMPs. RPA was performed as described in material and methods.

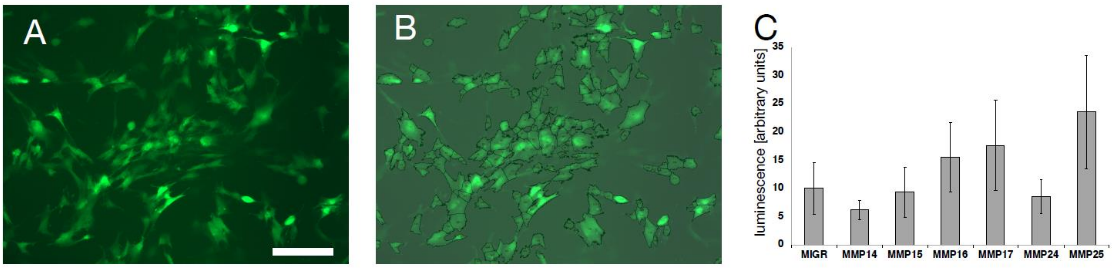

Figure S2. Expression of enhanced green fluorescent protein (EGFP) in transduced astrocytes. EGFP was expressed under transcriptional control of an IRES and was densitometrically measured in astrocyte cultures. (**A**) EGFP fluorescence was observed in all transduced astrocytes at different intensity. (**B**) Astrocytes were automatically detected by the imaging software Fiji and mean fluorescence intensity was measured per cell. (**C**) Mean luminescence of the different astrocyte cultures revealed comparable fluorescence intensity. Scale bar in A: 200 μm.

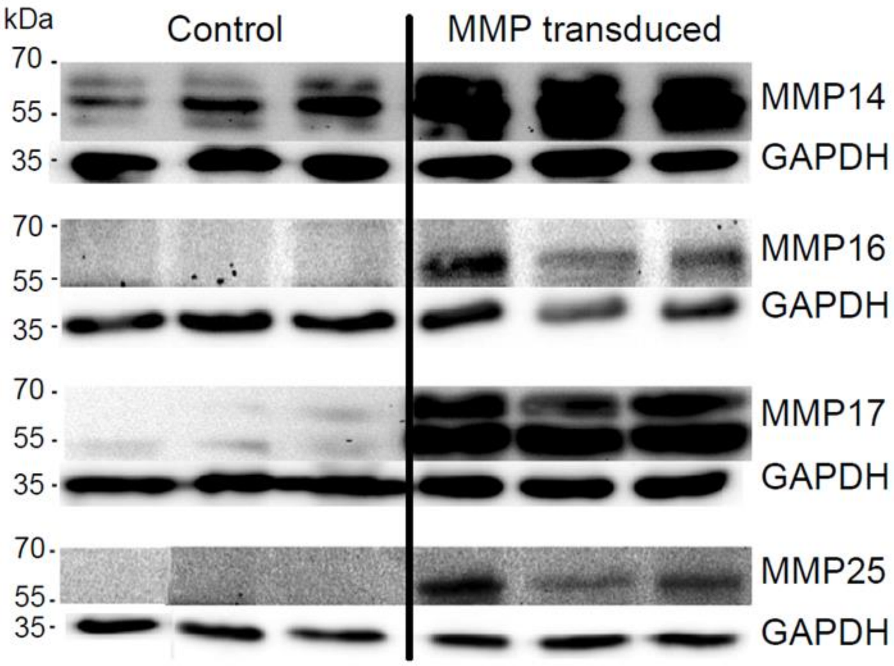

Figure S3. Expression of human MMP protein in transduced astrocytes. 50 μg of protein were analysed by Western blot using the appropriate primary antibody for the respective MMP. No expression was observed in control astrocytes and specific bands were observed for MMPs 14, 16, 17 and 25. Several antibodies against MMPs 15 and 24 did not produce specific bands with the respective astrocyte protein extracts. The lower part of each panel shows GAPDH which was used as a loading control.

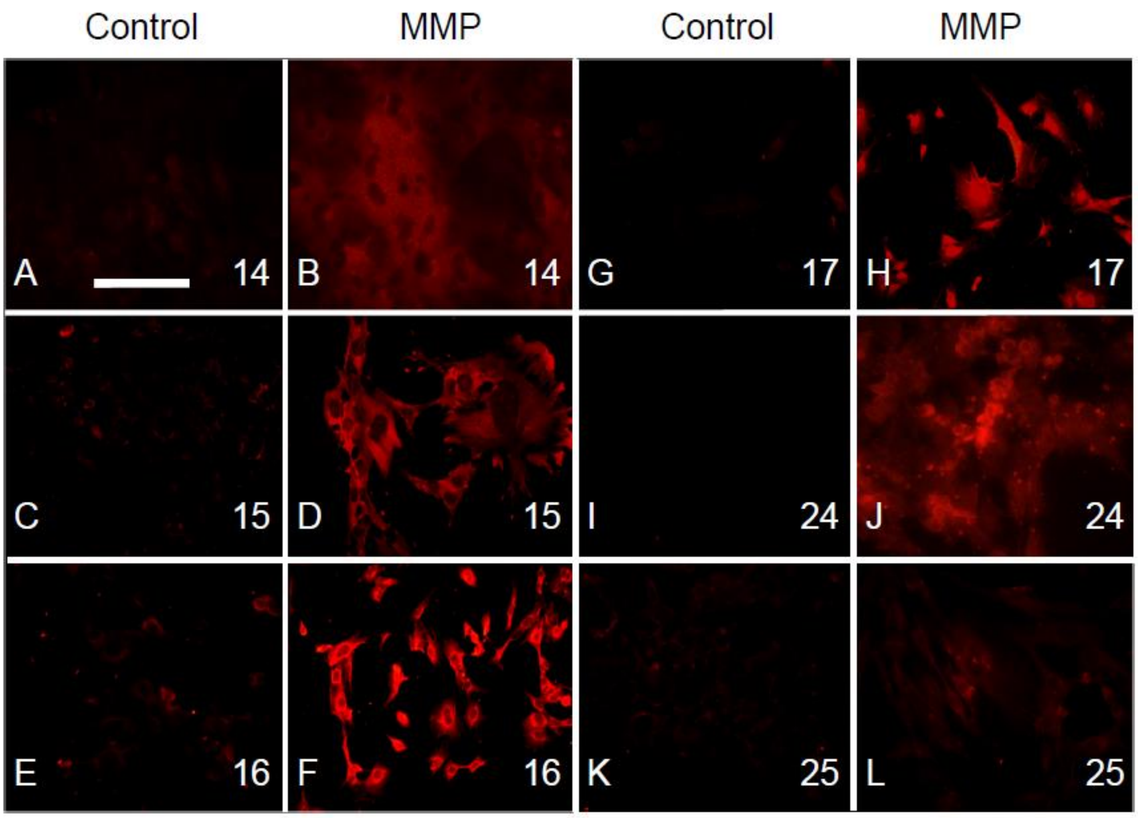

Figure S4. Expression of MT-MMP proteins in transduced astrocytes. Immunofluorescence revealed the presence of the respective human MT-MMPs on the cell membrane or in the cytoplasm of the transduced astrocytes. No immunoreactivity was observed in control astrocytes. Numbers in the lower right corner indicate the respective MMP (**A**, **B** MMP14, **C**, **D** MMP15, **E**, **F** MMP16, **G**, **H** MMP17, **I**, **J** MMP24, **K**, **L** MMP25). Scale bar in A: 100 µm in all figures except 200 µm in E and F.

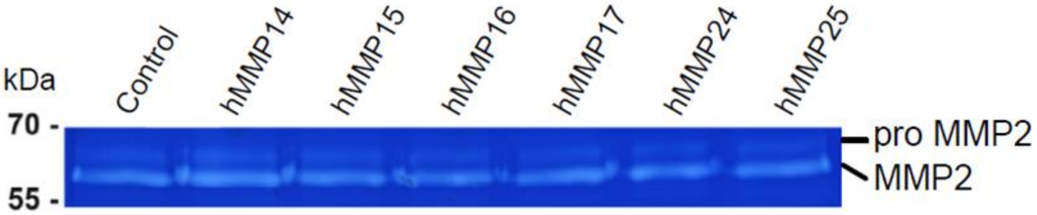

Figure S5. Gelatin zymography. Equal numbers of astrocytes were cultured for two days in serum-free medium. Culture supernatants were collected and 20 µL was separated in a 10% polyacrylamide gel containing 0,1% gelatin. Following separation, the gelatinases were activated and the gel incubated for 24 h at 37 °C. The larger portion of MMP2 in supernatants of primary astrocytes corresponds to activated MMP2 (lower band) while only low amounts of proMMP2 are present (upper band). There was no difference between control and MMP-transduced astrocytes with regard to activation of MMP2.

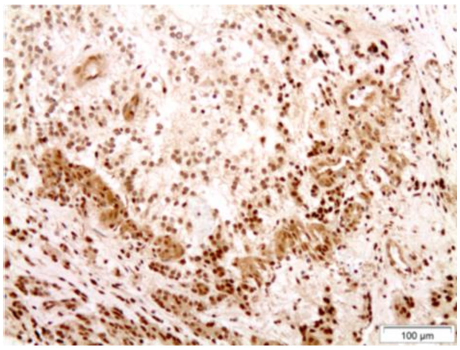

A

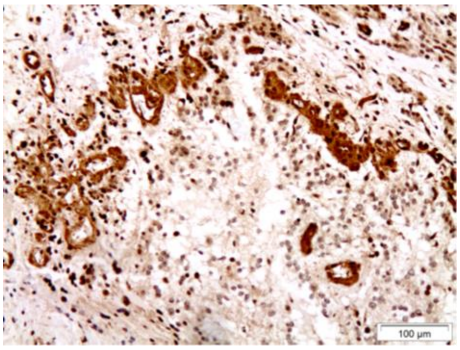

B

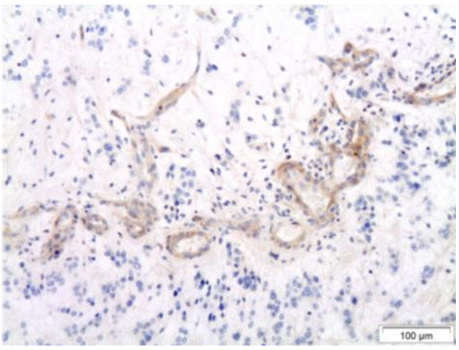

C

Figure S6: Immunohistochemical staining of MT-MMPs in pilocytic astrocytoma reaveled expression of (**A**) MMP14, (**B**) MMP16 and (**C**) MMP17 mainly in the endothelia of prominent vessel proliferations. Immunoreactivity for MMP14 and MMP16 was also observed in nuclei of tumour cells (bar: 100 µm).

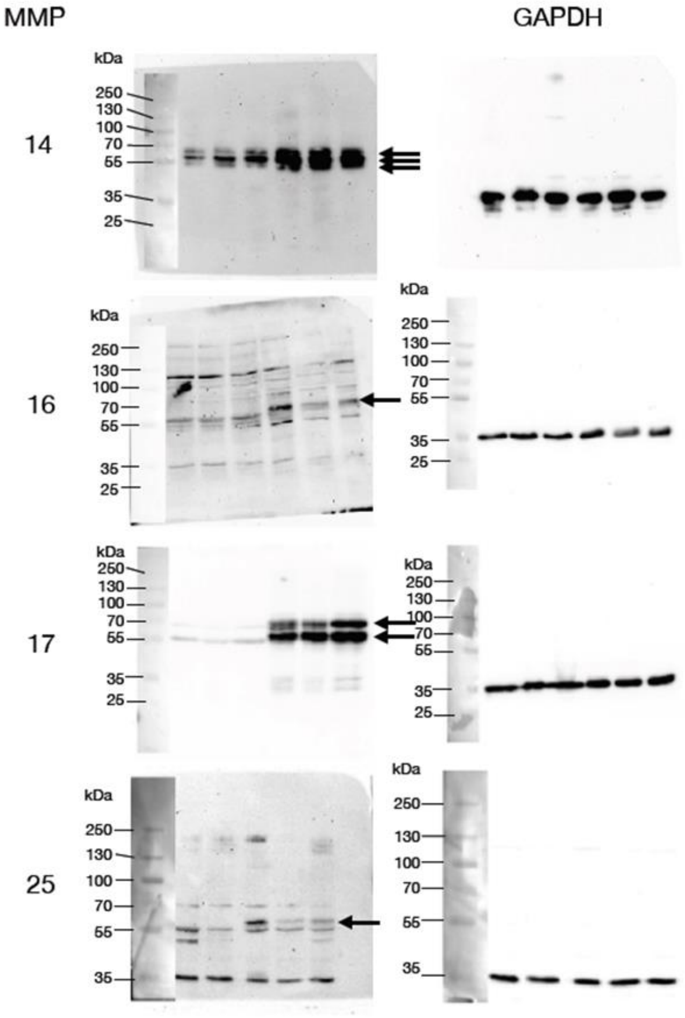

Figure S7: Detailed information about western blot in Figure S3.

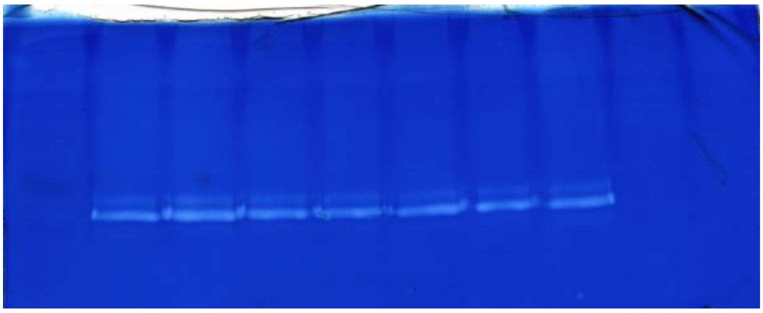

Figure S8: Detailed information about Figure S5.

Table S1: Primers and probe length for the human MT-MMP Probe Set.

Probe	Protected Base Pairs	5′ Primer	3′ Primer
MMP14	170	ccccaccaagatgccccctcaacc	ttcctcacccgccagaaccag
MMP15	202	gcccggccgcccttcaacc	ggacgcagagcagcagcagcagtg
MMP16	213	caaggattttatgggctgtga	agtatgtggcggggtgttcctt
MMP17	237	ccccagggcccggactctcg	caaactgctgcatggctgtgat
MMP24	256	ggcgacagctccccatttg	gcttcagcttgaagttgtgcgtctcc
MMP25	284	gcgatggcctgcagcaactctatg	ggcggcctgcaccaccctcacttg

© 2020 by the authors. Licensee MDPI, Basel, Switzerland. This article is an open access article distributed under the terms and conditions of the Creative Commons Attribution (CC BY) license (http://creativecommons.org/licenses/by/4.0/).
